# Dispersal Range of *Anopheles sinensis* in Yongcheng City, China by Mark-Release-Recapture Methods

**DOI:** 10.1371/journal.pone.0051209

**Published:** 2012-11-30

**Authors:** Qiyong Liu, Xiaobo Liu, Guangchao Zhou, Jingyi Jiang, Yuhong Guo, Dongsheng Ren, Canjun Zheng, Haixia Wu, Shuran Yang, Jingli Liu, Hongsheng Li, Huazhong Li, Qun Li, Weizhong Yang, Cordia Chu

**Affiliations:** 1 Department of Vector Biology and Control, State Key Laboratory for Infectious Diseases Prevention and Control, National Institute for Communicable Disease Control and Prevention, Chinese Center for Disease Control and Prevention, Beijing, China; 2 WHO Collaborating Centre for Vector Surveillance and Management, Beijing, China; 3 Yongcheng Center for Disease Control and Prevention, Yongcheng, Henan, China; 4 Public Health Emergency Center, Chinese Center for Disease Control and Prevention, Beijing, China; 5 Department of Infectious Diseases Control and Prevention, Chinese Center for Disease Control and Prevention, Beijing, China; 6 China CDC Key Laboratory of Surveillance and Early-Warning on Infectious Disease, Chinese Center for Disease Control and Prevention, Beijing, China; 7 Centre for Environment and Population Health, Griffith University, Nathan, Queensland, Australia; Instituto de Higiene e Medicina Tropical, Portugal

## Abstract

**Background:**

Studying the dispersal range of *Anopheles sinensis* is of major importance for understanding the transition from malaria control to elimination. However, no data are available regarding the dispersal range of *An. sinensis* in China. The aim of the present study was to study the dispersal range of *An. sinensis* and provide the scientific basis for the development of effective control measures for malaria elimination in China.

**Methodology/Principal Findings:**

Mark-Release-Recapture (MRR) experiments were conducted with 3000 adult wild *An. sinensis* in 2010 and 3000 newly emerged wild *An. sinensis* in 2011 in two villages of Yongcheng City in Henan Province. Marked *An. sinensis* were recaptured daily for ten successive days using light traps. The overall recapture rates were 0.83% (95% CI, 0.50%∼1.16%) in 2010 and 1.33% (95% CI, 0.92%∼1.74%) in 2011. There was no significant difference in the recapture rates of wild *An. sinensis* and newly emerged *An. sinensis*. The majority of *An. sinensis* were captured due east at study site I compared with most in the west at study site II. Eighty percent and 90% of the marked *An. sinensis* were recaptured within a radius of 100 m from the release point in study site I and II, respectively, with a maximum dispersal range of 400 m within the period of this study.

**Conclusions/Significance:**

Our results indicate that local *An. sinensis* may have limited dispersal ranges. Therefore, control efforts should target breeding and resting sites in proximity of the villages.

## Introduction

The global malaria elimination campaign is an ambitious goal [1]–[9]. Every step in the chain of transmission of malaria would be need to be a target for successful implementation of global elimination of malaria [4], [5], [10], [11]. In recent years, it is recognized that local outbreaks of malaria might result from incorrect control measures stemming from inadequate understanding of the ecological characteristics of the dominant vectors [12], environmental changes, such as global climate changes [13]–[20], or from drug and insecticides resistance [21]. Therefore, management of ecological habitats of the predominant malaria vectors in a region are of great significance for malaria control, via optimal allocation of resources [22], [23]. Today, malaria is not as serious as two decades ago in China [3], [24]. However, outbreaks occur when malaria cases are introduced to an area where malaria vectors are established and other local conditions favor transmission. At present, China has entered the critical period in the process of eliminating malaria according to WHO standards for global malaria eradication campaign. As the principal vector of *Plasmodium vivax* malaria [25], the species of *Anopheles sinensis* (Wiedemann, 1828) is distributed in most provinces in China. The dispersal range of *An. sinensis* in real rural villages can provide key reference data for epidemiological surveys of malaria cases. In addition, it can also provide the basis for the determination of the vector control range and for prevention of the emergence and spread of secondary cases. Therefore, the study of dispersal ranges of *An. sinensis* is an important factor to detect potential source of infection, cut off the route of transmission, and further ensure the successfully implementation of malaria elimination in China by 2020 [26].

The dispersal of mosquito vectors, to find mates, resting sites, oviposition sites, blood meals, and nectar sources, plays a major role in the transmission of malaria [27]. Mark-release-recapture (MRR) technique can be applied for estimating survival, cohort specific dispersal, gonotrophic cycle, and population density of mosquitoes. These population attributes are important for mosquito-borne disease control programs plan. Dispersal and survival are of considerable importance in studying the ecology of *Anopheles* mosquitoes [28]. Currently, MRR technique has been widely used in *Anopheles* species [29]–[34]. However, deficiencies in understanding of dispersal ranges of *An. sinensis* would impede development of effective management programmes to local outbreaks of malaria due to imported cases. In addition to dispersal, survival rates of adult *An. sinensis* plays a key role in malaria transmission and in the calculation of vectorial capacity [35], [36]. Survival of adults depends on many factors including larval and adult nutrition, temperature, predation, and genotype.

The planning of management programs for vector control in epidemic focus of malaria in China requires accurate information of dispersal range and survival of *An. sinensis*. However, limited data are available on the dispersal range and survival of *An. sinensis* in the real villages in China. Therefore, two field studies were conducted to examine the dispersal range and survival rate of *An. sinensis* in Yongcheng City of Henan Province where *P. vivax* malaria is unstable transmission. The objective was to provide a scientific basis for designing control strategies and tactics for malaria elimination in China.

## Methods

### Study area

The present study was conducted in two villages of Yongcheng City characterized by different levels of historical incidence of *P. vivax* malaria. These included a high risk village study site I (N 33°45.023′, E 116°13.151′, southern part of Yongcheng City with an average annual incidence rate >100/100,000), and an intermediate risk village study site II (N 33°52.492′, E 116°28.458′, middle part of Yongcheng City with an average annual incidence rate 10∼100/100,000) [37]. The distance between the two study sites is approximately 60 km. Besides the difference in the level of the historical incidence of *P. vivax* malaria, other differences between the two studied villages are as follows: First, study site I is adjacent to Guoyang County; study site II is neighbouring to Suixi County. Guoyang and Suixi County are unstable regions of *P. vivax* malaria in Anhui Province. Second, water-body distributions and appropriate breeding sites on *An. sinensis* larvae in study site II are more numerous than those in study site I. Third, the population of animal hosts in study site II was larger than that of study site I during the study period.

Most of the area is a plain at 33 meters altitude above sea level. The range of annual rainfall is between 556.2 mm and 1,648.9 mm, and most rainfall is peak in period of June-September [37]. The primary cultivated crops in the area include wheat, soybean and corn. The climate is warm temperate from May to October, and the average annual temperature is 14.3°C. The human dwellings in these two villages are made of bricks. The average family size was two person per house, together with their chickens, dogs and few other livestock. During summer, most of local residents tend to sleep outdoors [38]. With active cooperation of the villagers, the present mark-release-recapture studies were conducted with wild collected *An. sinensis* in study site I in 2010 and with newly emerged *An. sinensis* in study site II in 2011.

### Effect of marking with fluorescent powder on the survivorship of *An. sinensis*

Prior to the field study, the effect of marking *An. sinensis* with fluorescent pigment on the mortality rate was studied in the laboratory of the Chinese Center for Disease Control and Prevention (China CDC). A group of three-day-old *An. sinensis* (30 males and 30 females) was used and aspirated into a waxed, 200 mm diameter custom made cylinder with gauze tops, gauze bottoms and metal bracket. The powder for dusting was Day-Glo fluorescent pigment (Day-Glo Color Corp., Cleveland, Ohio, USA). A 5 ml syringe with a 22 gauge needle was used as a powder atomization device. Adults were manually aspirated and counted into the cylinder, and then the entrance of the cylinder was closed. The syringe which filled with fluorescent pigment was pushed very quickly to produce atomizing. After 30 minutes, all the 60 *An. sinensis* of the experimental group were marked with green fluorescent pigment. Thereafter, all marked *An. sinensis* were sprayed with distilled water 3 times a day with a small sprayer in order to simulate the effect of rainfall on the fluorescent pigment of the body surface of *An. sinensis*. We also set a control group of three-day-old *An. sinensis* (30 males and 30 females) without marking with fluorescent pigment. All mosquitoes were provided with 10% sucrose daily and held at 28°C and a relative humidity of 70∼80%. The number of live mosquitoes was counted daily for 14 days and marked *An. sinensis* in the experimental group were observed under a dissecting microscope in order to observe the existence of fluorescent powder on the body surface of *An. sinensis*.

### Adult capture and larval rearing

In study site I, wild adult *An. sinensis* were collected from a bovid shed and a sheepfold in Lizhai township of Yongcheng City with aspirators during the period of activity peak of *An. sinensis* in a day. All *An. sinensis* were collected within three days, placed into mosquito cages (45 cm ×45 cm ×45 cm) and then transported to a laboratory in Yongcheng CDC. In study site II, it was difficult to collect adult *An. sinensis* because of a severe drought. Therefore, larvae and pupae of *An. sinensis* were collected from the breeding sites. Three-day old newly emerged *An. sinensis* was transferred to a large custom made cylinder mentioned above, and ready to mark [39]. Both published literatures and local CDC staff observations showed that *An. sinensis* was the sole vector of *P. vivax* malaria in Yongcheng City [24], [37], [38], [40]. Prior to marking and releasing, some wild captured and newly emerged *An. sinensis* were also sampled and identified to species level by ribosomal DNA PCR assay [41] to insure that only *An. sinensis* was released.

### Marking and releasing

The first mark-release-recapture experiment took place from 14 to 23 October 2010, from a release point located at the edge of the study site I. The second mark-release-recapture experiment took place from 13 to 22 October 2011, with a release point located at a road crossing of the center of study site II [37]. The release time was at 19∶00 in 2010 and 2011. The female-to-male ratios of marked *An. sinensis* in these two years were about 1∶1. Before the marked *An. sinensis* were released, the geographical coordinates of sheep sheds, cattle sheds, pig pens, chicken sheds and potential breeding sites were recorded using hand-held GPS. One hundred and fifty male and female adult *An. sinensis* were marked at a time with green fluorescent pigment. One day later, all marked *An. sinensis* were transported to the release point. The entrance of the cage was opened slowly to allow the marked *An. sinensis* to fly out freely. The mosquitoes that seemed exhausted and did not fly out were counted, and their numbers were subtracted from the total marked *An. sinensis*.

### Recapturing and identification

To recapture the marked *An. sinensis* individuals, six light traps we set up in four directions (east, south, west, and north) and a light trap we set up at the release point at both study sites during the two years. In study site I, the light traps were set within courtyards in each direction with the distance 50, 100, 200, 300, 400, 500 meters, respectively. In total, twenty-five light traps were operated. In study site II, additional four light traps were added to sheepfolds which located at 50 m from the release site, one in each direction. Total twenty-nine light traps were operated in study site II. The light traps were set mainly in sheep folds within these distances so as to improve the recapture rate of marked *An. sinensis*. However, they were set in the courtyard if no sheep folds existed in these ranges.

The light trap at the release point was set up from 21∶00 to 06∶00 in the first day. From the second day, these light traps were set up from 18∶00 to 06∶00 for 9 successive nights after release in both study sites (I & II). Marked *An. sinensis* were recaptured in the same day after releasing. Some key meteorological parameters (temperature, relative humidity, precipitation, wind velocity and direction) were recorded during the study period. Temperature (°C) and relative humidity (%) were recorded from a weather website in China (http://www.weather.com.cn). Ambient outdoor air temperature and relative humidity of each collection hour was recorded using a WS-1 thermo-hygrometer device (Tianbayiqi Corp., Tianjin, China). All captured mosquitoes were killed by ether and morphologically identified into mosquito species. Then all the *Anopheles* mosquitoes were selected and examined for the presence or absence of fluorescent pigment under dissecting microscope.

### Ethics statement

The experimental protocols were approved by the Ethical Committee of National Institute for Communicable Disease Control and Prevention, Chinese Center for Disease Control and Prevention [42]. Although the release of *An. sinensis* temporally increased local mosquito populations in the two study villages, the experiment posed very low risk of public health because the malaria is about to be eliminated and no *Plasmodium* vivax was detected in *An. sinensis* of Yongcheng City in recent years. In addition, all released wild and newly emerged *An. sinensis* were from the local habitat. Verbal consent was obtained from all the heads of household to permit mosquito collection from their houses and livestock sheds prior to the procedure. The Ethical Committee of National Institute for Communicable Disease Control and Prevention, China CDC reviewed and approved the consent procedure. Permission was also obtained from the Municipal Health Bureau and Center for Disease Control and Prevention in Yongcheng City.

### Statistical methods

Recapture rates were calculated as the number of marked *An. sinensis* recaptured over the total number of originally released. The mortality rate between marked *An. sinensis* and untreated *An. sinensis* in the laboratory, and wild *An. sinensis* and newly emerged *An. sinensis* were compared by Chi-square analysis. Daily survival rates were estimated by fitting a linear regression model of logarithm number of recaptures against calendar day after releasing, assuming that the daily probability of survival was constant throughout the year. The calculation was done by regressing the number of recaptured *An. sinensis* transformed into ln (y+1) as a function of time in days post-release. Then, the daily survival rate was calculated as the antilogarithm of the regression coefficient [30], [43], [44]. Statistical analysis was carried out using SPSS software (Version 11.5 for Microsoft Windows, SPSS Inc., Chicago, USA).

## Results

### The effect of fluorescent pigment on the survivorship of *An. sinensis* in the laboratory

The research shows that there was no difference in mortality rates between marked and unmarked *An. sinensis* in the laboratory (χ^2^ = 3.12, P>0.05). During the 14 day observation and assay period, fluorescent pigment on the body surface of marked *An. sinensis* was detected under a dissecting microscope in females and males in all replicates. Based on the findings mentioned above, fluorescent pigments may have little effect on the survivorship of *An. sinensis* if its sprayed correctly and could be used to conduct MRR experiment [32], [45].

### Species identification

During the study period, prior to mark and release, 50 wild captured and 50 newly emerged anopheline mosquitoes were identified to species by ribosomal DNA PCR assay [46], and the results revealed that all anopheline mosquitoes examined belonged to *An. sinensis*.

### Release and recapture rate

In study site I, 3000 wild *An. sinensis* (1500 male, 1500 female ) were released, 25 of which were recaptured, corresponding to a recapture rate of 0.83% (95% CI, 0.50%∼1.16%). In study site II, 3000 newly emerged *An. sinensis* (1500 male, 1500 female ) were released, 40 of which were recaptured, corresponding to a recapture rate of 1.33% (95% CI, 0.92%∼1.74%) (Table 1). There was no significant difference in the recapture rates of wild *An. sinensis* and newly emerged *An. sinensis* (χ^2^ = 3.499, P>0.05) though more marked *An. sinensis* were recaptured in study site II.

**Table 1:** Dispersal range of recaptured marked *An. sinensis* according to the day after release in 2010 and in 2011.

Year	Recapture date	No. of recaptured marked *An. sinensis*							Total (%)
		0 m	50 m	100 m	200 m	300 m	400 m	500 m	
2010	10/14	0	0	1	0	0	0	0	1 (4.0)
	10/15	0	2	12	0	0	0	0	14 (56.0)
	10/16	1	2	1	1	1	2	0	8 (32.0)
	10/17	0	1	0	0	0	0	0	1 (4.0)
	10/18	0	0	0	0	0	0	0	0 (0.0)
	10/19	0	0	0	0	0	0	0	0 (0.0)
	10/20	0	0	0	0	1	0	0	1 (4.0)
	10/21	0	0	0	0	0	0	0	0 (0.0)
	10/22	0	0	0	0	0	0	0	0 (0.0)
	10/23	0	0	0	0	0	0	0	0 (0.0)
	Total (%)	1 (4.0)	5 (20.0)	14 (56.0)	1 (4.0)	2 (8.0)	2 (8.0)	0 (0.0)	25 (100.0)
	Recapture rate (%)	0.03	0.17	0.47	0.03	0.06	0.06	0.00	0.83
2011	10/13	5	2	0	0	0	0	0	7 (17.5)
	10/14	4	4	0	0	0	0	0	8 (20.0)
	10/15	0	3	1	0	0	0	0	4 (10.0)
	10/16	0	0	3	0	0	0	0	3 (7.5)
	10/17	0	2	0	1	0	0	0	3 (7.5)
	10/18	0	0	2	1	0	0	0	3 (7.5)
	10/19	0	1	1	1	0	0	0	3 (7.5)
	10/20	0	2	0	0	1	0	0	3 (7.5)
	10/21	0	1	1	0	0	0	0	2 (5.0)
	10/22	0	4	0	0	0	0	0	4 (10.0)
	Total (%)	9 (22.5)	19 (47.5)	8 (20.0)	3 (7.5)	1 (2.5)	0 (0.0)	0 (0.0)	40 (100.0)
	Recapture rate (%)	0.30	0.63	0.27	0.10	0.03	0.00	0.00	1.33

In study site I, all the recaptured marked *An. sinensis* were females and 6 out of 25 recaptured *An. sinensis* were engorged. In study site II, 37 out of 40 recaptured *An. sinensis* were females, and 9 out of 37 recaptured marked *An. sinensis* were engorged. The female-to-male ratio of recaptured marked *An. sinensis* in study site II was 37: 3.

### Dispersal ranges and directions

In study site I, one marked *An. sinensis* was recaptured at the release point, and 5, 14, 1, 2 and 2 marked *An. sinensis* were recaptured at 50, 100, 200, 300 and 400 m from the release point, respectively. Eighty percent of marked *An. sinensis* were recaptured within a radius of 100 m from the release point. The maximum distance traveled was 400 m, where 2 *An. sinensis* were recaptured. In study site II, 9 marked *An. sinensis* were recaptured at the release point, 19, 8, 3, and 1 marked *An. sinensis* were recaptured at 50, 100, 200 and 300 m from the release point, respectively. Ninety percent of marked *An. sinensis* were recaptured within a radius of 100 m from the release point. The dispersal ranges of marked *An. sinensis* per day after release are shown in Table 1.

In study site I, 13 marked *An. sinensis* were recaptured to the east of the release point, 9 marked *An. sinensis* were recaptured to the south, and 2 marked *An. sinensis* to the north. In study site II, 14 marked *An. sinensis* were recaptured to the south of the release point, 16 marked *An. sinensis* to the west and 1 marked *An. sinensis* to the north. There was a significant difference in the recapture rates of release marked *An. sinensis* in different directions (χ^2^ = 30.016, P<0.01). The majority of *An. sinensis* were recaptured east in study site I while this was true for the west in study site II. The number of recaptured *An. sinensis* in distinct directions of two villages is shown in Table 2.

**Table 2:** Numbers of recaptured marked *An. sinensis* in distinct directions in two villages of Yongcheng City.

Year	Direction	No. of *An. sinensis* recaptured							Total (%)	Recapture rate (%)
		0 m	50 m	100 m	200 m	300 m	400 m	500 m		
2010	Release point	1	–	–	–	–	–	–	1 (4.0)	0.03
	East	–	3	7	1	0	2	0	13 (52.0)	0. 43
	South	–	2	7	0	0	0	0	9 (36.0)	0.30
	West	–	0	0	0	0	0	0	0 (0.0)	0.00
	North	–	0	0	0	2	0	0	2 (8.0)	0.07
	Total (%)	1 (4.0)	5 (20.0)	14 (56.0)	1 (4.0)	2 (8.0)	2 (8.0)	0 (0.0)	25 (100.0)	0.83
2011	Release point	9	–	–	–	–	–	–	9 (22.5)	0.30
	East	–	0	0	0	0	0	0	0 (0.0)	0.00
	South	–	11	1	1	1	0	0	14 (35.0)	0.47
	West	–	8	7	1	0	0	0	16 (40.0)	0.53
	North	–	0	0	1	0	0	0	1 (2.5)	0.03
	Total (%)	9 (22.5)	19 (47.5)	8 (20.0)	3 (7.5)	1 (2.5)	0 (0.0)	0 (0.0)	40 (100.0)	1.33

### Survival rate

**Figure 1:**
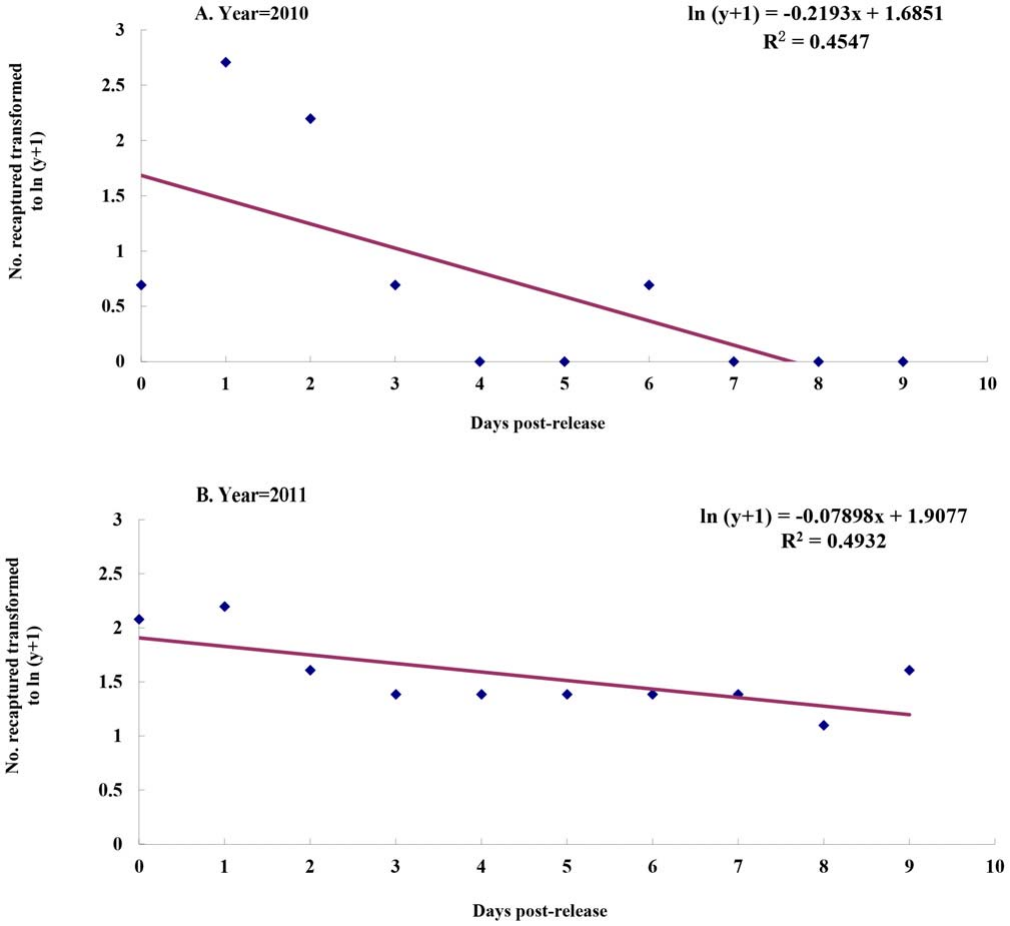
Regression of the daily number (ln y+1) of marked *An. sinensis* recaptured in days post-release in 2010 and in 2011.

The log-transformed number of recaptured *An. sinensis* decreased significantly as a linear function of time in days post-release (Fig. 1). In study site I, the regression equation was ln (y+1)  = −0.2193×+1.6851, and the coefficient of determination R^2^ was 0.4547. Then daily survival rate, calculated as the antilogarithm of the regression coefficient [−0.2193 (95% CI, −0.05286∼ −0.3857)], was 0.8031 (95% CI, 0.6799∼0.9485). In study site II, the regression equation was ln (y+1)  = −0.07898×+1.9077, and the coefficient of determination R^2^ was 0.4932. Then the daily survival rate, calculated as the antilogarithm of the regression coefficient [−0.07898 (95% CI, −0.0352∼ −0.1345)], was 0.9240 (95% CI, 0.8742∼0.9654) [30].

### The meteorological factor

There was no significant difference in climatic conditions between two study sites during the study period. The range of the temperature varied from 11.5∼27.0°C in study site I and 9.0∼26.0°C in study site II. No rainfall was recorded of the study period during either year. The wind velocity was low and the main wind direction in study site I was west wind while not regular in study site II during the study period. Other relevant meteorological parameters are shown in Table 3.

**Table 3:** The meteorological parameters during the study period in two villages of Yongcheng City.

Year	Recapture date	Temperature (°C)	Relative humidity (%)	Precipitation (mm)	Wind velocity (m/s)	Wind direction
2010	10/14	14.7∼23.4	35∼75	0	0.3∼3.4	W
	10/15	13.5∼23.7	39∼81	0	0.0∼2.2	NE
	10/16	11.7∼27.0	16∼86	0	0.3∼3.5	SE
	10/17	12.4∼24.1	38∼81	0	0.2∼3.2	W
	10/18	15.0∼26.5	24∼90	0	0.2∼3.8	SW
	10/19	13.4∼22.7	57∼90	0	0.2∼2.7	NE
	10/20	12.3∼19.8	63∼86	0	0.9∼3.4	N
	10/21	11.5∼22.5	36∼92	0	0.1∼3.8	E
	10/22	12.2∼23.8	33∼82	0	0.3∼3.5	E
	10/23	13.3∼24.3	39∼90	0	0.3∼5.0	E
2011	10/13	11.0∼22.0	79∼98	0	0.0∼2.0	N
	10/14	9.0∼22.0	32∼97	0	0.0∼2.0	NW
	10/15	13.0∼21.0	28∼70	0	0.0∼2.0	N
	10/16	12.0∼22.0	17∼66	0	0.0∼2.0	N
	10/17	12.0∼26.0	28∼87	0	0.0∼2.0	Not regular
	10/18	13.0∼25.0	25∼94	0	1.0∼3.0	SE
	10/19	16.0∼25.0	31∼91	0	0.0∼2.0	Not regular
	10/20	14.0∼22.0	48∼90	0	0.0∼2.0	Not regular
	10/21	14.0∼21.0	68∼90	0	0.0∼1.0	W
	10/22	13.0∼23.0	83∼100	0	0.0∼1.0	E

Temperature (°C) and relative humidity (%) were recorded from a weather website in China (http://www.weather.com.cn). Ambient outdoor air temperature and relative humidity of each collection hour was recorded using a WS-1 thermo-hygrometer device (Tianbayiqi Corp., Tianjin, China).

## Discussion

The present study showed a recapture rate of marked *An. sinensis* of 0.83% and 1.33% in study site I and II, respectively. These recapture rates are affected by the experimental design, the source and species of mosquitoes, the resting and oviposition sites, the availability of host blood meals, the age structure of mosquitoes [47], configuration of dispersal area [48], local geography and topography, mosquito collection methods [49], [50]. Reisen et al reported a female recapture rate of female *An. culicifacies* was 8.0%, with a 5.9% rate for males, captured mainly in houses and cattle houses [51]. Jaal et al reported that only 3 out of 8 species of *Anopheline* mosquitoes were recaptured with the recapture rates of 3.42% for *An. lesteri paraliae*, 1.19% for *An. subpictus*, and 0.97% for *An. vagus* [45]. Kligler reported that *An. sacharovi* flew over 13 km from their larval habitat, and *An. freeborni* flies 42 km from the release site to find a place for passing the winter [52].

It is reported that light traps can be used as an alternative to human biting catches of *An. sinensis* in the study area and is a promising tool for sampling malaria vector populations [42]. Therefore, light traps were used to recapture the marked *An. sinensis* in this study. These light traps were operated within the courtyards to avoid a sample bias which may affect the dispersal of *An. sinensis*. Using this method, the effective attraction radius for the light traps was shortened because walls blocked the passage of light. In the first day, light trap at the release point was opened at 21∶00 to ensure that marked mosquitoes had an opportunity to leave the release area.

Both females and males were captures in the light traps. For the marked *An. sinensis*, more females than males were recaptured in general. This phenomenon could be due to the light traps becoming more efficient at capturing females when they seeking blood meals.

The present study was similar to a MRR experiment with *An. sinensis* in the northern part of Gyeonggi-do, Korea [48]. However, the recapture rate in the present study was slightly lower than that of Gyeonggi-do' study (1.52%). The differences in recapture rate could be explained by the difference in mosquito collection methods. Light traps of their studies were set mainly in cattle sheds. In contrast, only a small proportion of light traps were set in cattle sheds because of the relatively small number of livestock in the study villages. However, recapture rates in MRR experiments involving anopheline or culicine mosquitoes are often less than 1% [45].

Relying on newly emerged female anopheline mosquitoes could improve recapture rates and provide more data for MRR studies [39], [53]. In this study, wild captured *An. sinensis* were released in study site I while newly emerged *An. sinensis* were released in study site II. The reason why two sources of *An. sinensis* were employed is that newly emerged mosquitoes may improve the recapture rate compared to wild captured adults [54]. The relatively lower recapture rate in study site I (0.83%) may be partly attributed to the differences in the sources of released mosquitoes [53].

In this study, both male and female *An. sinensis* were released. In study site I, all the recaptured marked *An. sinensis* were females while 37 out of 40 recaptured marked *An. sinensis* were females in study site II. The reason why male *An. sinensis* were released was to add additional information to the recapture rate of each sex to the MRR experiment.

In this study, the longest range for setting light traps was 500 m, and this range is shorter than similar studies in Korea [48]. Based on our observations in the field, the distance between release point and edge of most of the villages was less than 400 meters in Yongcheng City. In addition, we studied the dispersal range in real rural villages rather than that in open uninhabited areas. Therefore, from the point of view of vector control targeted malaria elimination, epidemiological survey of detected and undetected cases when malaria occurred, the prevention of emergence and spread of secondary cases, 500 m as the maximum radius in the village could be considered an adequate range.

Previous research showed that the dispersal range of mosquitoes can mainly be influenced by local environmental characteristics rather than mosquito species. In this study, 80% and 90% of the marked *An. sinensis* were recaptured within a radius of 100 m from the release point in study site I and study site II, respectively. The furthest recapture ranges were 400 m and 300 m in study site I and study site II, respectively. The dispersal ranges in the present study were shorter than that of a study in Gyeonggi-do, Korea [48]. In their study, 37.1% marked *An. sinensis* were recaptured in light traps set at 1 km from the release point [48]. In Yongcheng City, the distance between release point and edge of most of the villages was less than 400 m. Farms were the principal habitat beyond 400 m and a few crops grow in the farm during the study period. It is possible that the diversity of obstacles posed by the irregular and dense structures in these two villages, associated with high availability of blood meals hosts and breeding sites within a radius of 400 m, constrained the dispersal of *An. sinensis*, where no mosquitoes flew beyond 400 m from the release point. Therefore, when a confirmed malaria case is reported during the critical period of *P. vivax* malaria elimination, emergency vector control activities should target *An. sinensis* larvae and adults within a 400 m radius of confirmed case, and 100 m is the key radius of the vector control activities.

Regarding the dispersal directions of marked *An. sinensis* in the present study, most of marked *An. sinensis* were recaptured in the east (13) and south (9) in study site I. In contrast, most of marked *An. sinensis* were recaptured in the west (16) and south (14) in study site II. Based on the field observations, there were more livestock sheds distributed in the east of study site I while more livestock sheds in the west of study site II. In addition, the main wind direction in study site I was west wind while not regular in study site II. However, structures, geography, wind velocity were similar between two villages during the study period.

The daily survival rate could be influenced by temperature, food availability, host destruction, predation by natural enemies (dragonfly, bat, bufonid, Gekko japonicus, spider, etc.), and other environment factors. Loss of marked mosquitoes was probably mainly due to migration out of the trapping area, loss of marking and death. All these would inflate the estimate of daily mortality of *An. sinensis*. Based on our laboratory experiment, fluorescent pigments may have little effect on the survivorship of *An. sinensis* if sprayed correctly during the 14 day observation and assay period. Fluorescent pigment on the body surface of marked *An. sinensis* can be detected under dissecting microscope in all replicates. In the field, we also observed recaptured *An. sinensis* could be 100% identifiability in the laboratory during the study period. In the present study, the estimated daily survival rates of *An. sinensis* were relatively low, thought this phenomenon was in line with other reported studies [24]. The relatively limited dispersal range of marked *An. sinensis* could be explained to some extent by the relatively low daily survival rates. Though the daily survival rates during the study period were not high, it is possible that a malaria outbreak would take place if source infection is introduced, ineffective vector control and high population susceptibility. Therefore, it poses a challenge to the implementation of malaria elimination in China by 2020.

Other potentially important aspects of MRR experiment were time of release, sampling intensity and weather conditions [50]. The release time of the present two MRR studies was at 19∶00. The reason why this time was selected as release time was that the density of *An. sinensis* begin to rise during these times in a day [37]. Weather conditions, especially heavy rains, have played an important role in the lower recapture rate of *An. saperoi* by restricting the movements of most of the released mosquitoes [30]. Fortunately, a favorable factor was that no rain happened during the study period in study site I and study site II. However, an unfavorable factor was that the temperature was relatively lower during the study period in these two years. This might exert reverse effect on the recapture rate and flight activity of marked *An. sinensis*.

This paper describes the first mark-release-recapture experiment using both wild and newly emerged *An. sinensis* to determine the recapture rate, dispersal range and survival rate of fluorescent pigment marked *An. sinensis* in two rural villages of Yongcheng City, a representative region of unstable *P. vivax* malaria transmission in the central part of China. The findings of our research could provide the scientific basis for the development of effective control measures for malaria elimination in China.

Care needs to be taken in interpreting the results of this study. First of all, there is no replication in this study. It was difficult to obtain *An. sinensis* because of the number of released *An. sinensis* adults and larvae were affected by severe drought in the two years. Therefore, the release sites, life history stages and trapping grids varied between years. These variations make direct comparisons of the results from 2010 and 2011 difficult. Secondly, constructions and physical barriers in the villages, such as houses and other buildings, may influence the dispersal of marked *An. sinensis*. Thirdly, the number of released *An. sinensis* in these MRR experiments was relatively small, and this may cause some impact on the recapture rates of marked *An. sinensis*. Fourth, the relationship between the recapture rate of marked *An. sinensis* and the meteorological conditions should be further analyzed by spatial analysis and GIS software could be used in similar studies in future. Finally, the weight of fluorescent pigment on the body of mosquito individuals was not considered in this study.

## References

[1] Kilama W, Ntoumi F (2009) Malaria: a research agenda for the eradication era. Lancet 374: 1480–1482. 19880004 10.1016/S0140-6736(09)61884-5

[2] Aguas R, White LJ, Snow RW, Gomes MG (2008) Prospects for malaria eradication in sub-Saharan Africa. PLoS One 3: e1767. 18335042 10.1371/journal.pone.0001767 PMC2262141

[3] Feachem RG, Sabot OJ (2007) Global malaria control in the 21st century: a historic but fleeting opportunity. JAMA 297: 2281–2284. 17519417 10.1001/jama.297.20.2281

[4] Das P, Horton R (2010) Malaria elimination: worthy, challenging, and just possible. Lancet 376: 1515–1517. 21035845 10.1016/S0140-6736(10)61551-6

[5] Feachem R, Sabot O (2008) A new global malaria eradication strategy. Lancet 371: 1633–1635. 18374409 10.1016/S0140-6736(08)60424-9

[6] Hsiang MS, Abeyasinghe R, Whittaker M, Feachem RG (2010) Malaria elimination in Asia-Pacific: an under-told story. Lancet 375: 1586–1587. 20452505 10.1016/S0140-6736(10)60350-9

[7] Roberts L, Enserink M (2007) Malaria. Did they really say ... eradication? Science 318: 1544–1545. 18063766 10.1126/science.318.5856.1544

[8] Butler D (2009) Initiative targets malaria eradication. Nature 462: 19. 19890296 10.1038/462017a

[9] Campbell CC (2009) Malaria control-addressing challenges to ambitious goals. N Engl J Med 361: 522–523. 19641210 10.1056/NEJMe0905159

[10] Alonso PL, Brown G, Arevalo-Herrera M, Binka F, Chitnis C, et al (2011) A research agenda to underpin malaria eradication. PLoS Med 8: e1000406. 21311579 10.1371/journal.pmed.1000406 PMC3026687

[11] Kappe SH, Vaughan AM, Boddey JA, Cowman AF (2010) That was then but this is now: malaria research in the time of an eradication agenda. Science 328: 862–866. 20466924 10.1126/science.1184785

[12] The malERA Consultative Group on Vector Control (2011) A research agenda for malaria eradication: vector control. PLoS Med 8: e1000401. 21311587 10.1371/journal.pmed.1000401 PMC3026704

[13] Haque U, Hashizume M, Glass GE, Dewan AM, Overgaard HJ, et al (2010) The role of climate variability in the spread of malaria in bangladeshi highlands. PLoS One 5: e14341. 21179555 10.1371/journal.pone.0014341 PMC3002939

[14] Paaijmans KP, Wandago MO, Githeko AK, Takken W (2007) Unexpected high losses of *Anopheles gambiae* larvae due to rainfall. PLoS One 2: e1146. 17987125 10.1371/journal.pone.0001146 PMC2063461

[15] Bouma MJ, Sondorp HE, van der Kaay HJ (1994) Climate change and periodic epidemic malaria. Lancet 343: 1440. 10.1016/s0140-6736(94)92569-0 7910922

[16] Hales S, Woodward A (2003) Climate change will increase demands on malaria control in Africa. Lancet 362: 1775. 10.1016/S0140-6736(03)14939-2 14654310

[17] Hales S, Woodward A (2005) Global climate change and malaria. Lancet Infect Dis 5: 258–259; author reply 259–260. 10.1016/S1473-3099(05)70092-X 15854878

[18] Goklany IM (2004) Climate change and malaria. Science 306: 55–57; author reply 55–57. 10.1126/science.306.5693.55 15459370

[19] Gething PW, Smith DL, Patil AP, Tatem AJ, Snow RW, et al (2010) Climate change and the global malaria recession. Nature 465: 342–345. 20485434 10.1038/nature09098 PMC2885436

[20] Hay SI, Cox J, Rogers DJ, Randolph SE, Stern DI, et al (2002) Climate change and the resurgence of malaria in the East African highlands. Nature 415: 905–909. 11859368 10.1038/415905a PMC3164800

[21] Yewhalaw D, Wassie F, Steurbaut W, Spanoghe P, Van Bortel W, et al (2011) Multiple insecticide resistance: an impediment to insecticide-based malaria vector control program. PLoS One 6: e16066. 21264325 10.1371/journal.pone.0016066 PMC3020220

[22] Gou GX, Li DF, Shang LY, Wang WX, Sui QL, et al (1998) The study on ecological habits of Anopheles sinensis in Guantang, Luyi county from 1971 to 1996. Chin J Vector Biol & Control 9: 133–134 (in Chinese)..

[23] Ferguson HM, Dornhaus A, Beeche A, Borgemeister C, Gottlieb M, et al (2010) Ecology: a prerequisite for malaria elimination and eradication. PLoS Med 7: e1000303. 20689800 10.1371/journal.pmed.1000303 PMC2914634

[24] Zhou SS, Huang F, Wang JJ, Zhang SS, Su YP, et al (2010) Geographical, meteorological and vectorial factors related to malaria re-emergence in Huang-Huai River of central China. Malar J 9: 337. 21092326 10.1186/1475-2875-9-337 PMC3003275

[25] Mueller I, Galinski MR, Baird JK, Carlton JM, Kochar DK, et al (2009) Key gaps in the knowledge of *Plasmodium vivax*, a neglected human malaria parasite. Lancet Infect Dis 9: 555–566. 19695492 10.1016/S1473-3099(09)70177-X

[26] Liu XB, Liu QY, Guo YH, Jiang JY, Ren DS, et al (2012) Random repeated cross sectional study on breeding site characterization of *Anopheles sinensis* larvae in distinct villages of Yongcheng City, People's Republic of China. Parasit Vectors 5: 58. 22444032 10.1186/1756-3305-5-58 PMC3323357

[27] Killeen GF, Knols BG, Gu W (2003) Taking malaria transmission out of the bottle: implications of mosquito dispersal for vector-control interventions. Lancet Infect Dis 3: 297–303. 12726980 10.1016/s1473-3099(03)00611-x

[28] Baber I, Keita M, Sogoba N, Konate M, Diallo M, et al (2010) Population size and migration of *Anopheles gambiae* in the Bancoumana Region of Mali and their significance for efficient vector control. PLoS One 5: e10270. 20422013 10.1371/journal.pone.0010270 PMC2858161

[29] Achee NL, Grieco JP, Andre RG, Rejmankova E, Roberts DR (2007) A mark release-recapture study to define the flight behaviors of *Anopheles vestitipennis* and *Anopheles albimanus* in Belize, Central America. J Am Mosq Control Assoc 23: 276–282. 10.2987/8756-971X(2007)23[276:AMSTDT]2.0.CO;2 17939506

[30] Fabian MM, Toma T, Tsuzuki A, Saita S, Miyagi I (2005) Mark-release-recapture experiments with *Anopheles saperoi* (Diptera: Culicidae) in the Yona Forest, northern Okinawa, Japan. Southeast Asian J Trop Med Public Health 36: 54–63. 15906642

[31] Achee NL, Grieco JP, Andre RG, Rejmankova E, Roberts DR (2005) A mark-release-recapture study using a novel portable hut design to define the flight behavior of *Anopheles darlingi* in Belize, Central America. J Am Mosq Control Assoc 21: 366–379. 16506561 10.2987/8756-971X(2006)21[366:AMSUAN]2.0.CO;2

[32] Tsuda Y, Takagi M, Suwonkerd W (2000) A mark-release-recapture study on the spatial distribution of host-seeking anophelines in northern Thailand. J Vector Ecol 25: 16–22. 10925793

[33] Tsuda Y, Takagi M, Toma T, Sugiyama A, Miyagi I (1999) Mark-release-recapture experiment with adult *Anopheles minimus* (Diptera: Culicidae) on Ishigaki Island, Ryukyu Archipelago, Japan. J Med Entomol 36: 601–604. 10534954 10.1093/jmedent/36.5.601

[34] Toure YT, Dolo G, Petrarca V, Traore SF, Bouare M, et al (1998) Mark-release-recapture experiments with *Anopheles gambiae s.l*. in Banambani Village, Mali, to determine population size and structure. Med Vet Entomol 12: 74–83. 9513942 10.1046/j.1365-2915.1998.00071.x

[35] Lines J, Whitty CJM, Hanson K (2007) Prospects for eradication and elimination of malaria: a technical briefing for DFID. London School of Hygiene and Tropical Medicine.

[36] Boni MF, Buckee CO, White NJ (2008) Mathematical models for a new era of malaria eradication. PLoS Med 5: e231. 19067482 10.1371/journal.pmed.0050231 PMC2586351

[37] Liu XB, Liu QY, Guo YH, Jiang JY, Ren DS, et al (2011) The abundance and host-seeking behavior of culicine species (Diptera: Culicidae) and *Anopheles sinensis* in Yongcheng city, People's Republic of China. Parasit Vectors 4: 221. 22115320 10.1186/1756-3305-4-221 PMC3267684

[38] Chow CY (1991) Malaria vectors in China. Chinese Journal of Entomology Special Publ: 67–79, (in Chinese).

[39] Midega JT, Mbogo CM, Mwnambi H, Wilson MD, Ojwang G, et al (2007) Estimating dispersal and survival of *Anopheles gambiae* and *Anopheles funestus* along the Kenyan coast by using mark-release-recapture methods. J Med Entomol 44: 923–929. 18047189 10.1603/0022-2585(2007)44[923:edasoa]2.0.co;2 PMC2705338

[40] Zhang HW, Su YP, Zhou GC (2007) Re-emerging malaria in Yongcheng city of Henan province. Chin J Vector Bio & Control 18: 42–44 (in Chinese)..

[41] Ma YJ, Qu FY, Xu JN (1998) Differentiation of *Anopheles sinensis* and *Anopheles anthropophagus* using a ribosomal DNA PCR assay. Acad J Sec Mil Med Univ 19: 237–239 (in Chinese)..

[42] Wang DQ, Tang LH, Gu ZC, Zheng X, Yang MN, et al (2012) Comparative evaluation of light-trap catches, electric motor mosquito catches and human biting catches of Anopheles in the Three Gorges Reservoir. PLoS One 7: e28988. 22235256 10.1371/journal.pone.0028988 PMC3250403

[43] Gillies MT (1961) Studies on the dispersion and survival of *Anopheles gambiae Giles* in East Africa, by means of marking and release experiments. Bulletin of Entomological Research 52: 99–127.

[44] Reisen WK, Aslamkhan M (1979) A release-recapture experiment with the malaria vector, *Anopheles stephensi Liston*, with observations on dispersal, survivorship, population size, gonotrophic rhythm and mating behaviour. Ann Trop Med Parasitol 73: 251–269. 496476 10.1080/00034983.1979.11687255

[45] Jaal Z, MacDonald WW (1992) A mark-release-recapture experiment with *Anopheles lesteri paraliae* in northwest Peninsular Malaysia. Ann Trop Med Parasitol 86: 419–424. 1463364 10.1080/00034983.1992.11812687

[46] Ma YJ, Qu FY, Xu JN, Zheng ZM (1998) Differentiation of *Anopheles sinensis* and *Anopheles anthropophagus* using a ribosomal DNA PCR assay. Acad J Sec Mil Med Univ 19: 237–239 (in Chinese)..

[47] Rawlings P, Curtis CF, Wickramasinghe MB, Lines J (1981) The influence of age and season on dispersal and recapture of *Anopheles culicifacies* in Sri Lanka. Ecol Entomol 6: 307–319.

[48] Cho SH, Lee HW, Shin EH, Lee HI, Lee WG, et al (2002) A mark-release-recapture experiment with *Anopheles sinensis* in the northern part of Gyeonggi-do, Korea. Korean J Parasitol 40: 139–148. 12325443 10.3347/kjp.2002.40.3.139 PMC2721040

[49] Muir LE, Kay BH (1998) *Aedes aegypti* survival and dispersal estimated by mark-release-recapture in northern Australia. Am J Trop Med Hyg 58: 277–282. 9546403 10.4269/ajtmh.1998.58.277

[50] Reisen WK, Lothrop HD, Lothrop B (2003) Factors influencing the outcome of mark-release-recapture studies with *Culex tarsalis* (Diptera: Culicidae). J Med Entomol 40: 820–829. 14765659 10.1603/0022-2585-40.6.820

[51] Reisen WK, Mahmood F, Parveen T (1980) *Anopheles culicifacies Giles*: a release-recapture experiment with cohorts of known age with implications for malaria epidemiology and genetical control in Pakistan. Trans R Soc Trop Med Hyg 74: 307–317. 7434425 10.1016/0035-9203(80)90089-9

[52] Kliger IJ (1932) The movements of anopheles at various seasons of the year with special reference to infected mosquitoes. Trans R Soc Trop Med Hyg 26: 73–78.

[53] Ejerctto A, Urbino CM (1951) Flight range of gravid and newly emerged Anopheles. Bull World Health Organ 3: 663–671. 14821773 PMC2554024

[54] Harrington LC, Buonaccorsi JP, Edman JD, Costero A, Kittayapong P, et al (2001) Analysis of survival of young and old *Aedes aegypti* (Diptera: Culicidac) from Puerto Rico and Thailand. J Med Entomol 38: 537–547. 11476334 10.1603/0022-2585-38.4.537

